# Exploring communities’ perceptions of the etiology of illnesses in newborns and young infants 0–59 days old in 4 counties in Kenya

**DOI:** 10.1371/journal.pone.0240852

**Published:** 2020-11-17

**Authors:** George Odwe, Charity Ndwiga, Chantalle Okondo, Pooja Sripad, Timothy Abuya, Charlotte E. Warren

**Affiliations:** 1 Population Council, Nairobi, Kenya; 2 Population Council, Washington, DC, United States of America; Johns Hopkins School of Public Health, UNITED STATES

## Abstract

**Background:**

Understanding communities’ beliefs about the causes of illnesses in sick young infants (SYIs) is key to strengthening interventions and improving newborn health outcomes. This study explored communities' perception of the etiology of illnesses in SYIs 0–59 days old in four counties in Kenya.

**Methods:**

We used an exploratory qualitative study design. Data were collected between August and September 2018 and involved 23 in-depth interviews with female caregivers aged 15-24years; 25 focus group discussions with female caregivers aged 15–18 years, 19–24 years and 25–45 years; and 7 focus group discussions with fathers aged 18–34 years and 35 or more years. Participants were purposely sampled, only those with SYIs 0–59 days old were eligible to participate. Data were analyzed using inductive thematic analysis framework approach.

**Results:**

Female caregivers and fathers attributed illnesses in SYIs 0–59 days old to natural (biomedical) and supernatural causes which sometimes co-existed. There were commonalities in perceived natural causes of illness in SYIs across sites, age groups and gender. Perceived natural causes of illness in SYIs include unfavorable environmental and hygiene conditions, poor maternal and child nutrition, and healthcare practices. Perceived supernatural causes of illness in SYIs such as ‘evil eyes’ were common across the four counties while others were geographically unique such as the belief that owls cause illnesses.

**Conclusion:**

Communities’ understanding of the etiology of illnesses in SYIs in the study settings overlapped between natural and supernatural causes. There is need for child health programmes to take into consideration communities’ beliefs and practices regarding disease and health to improve newborn health outcomes.

## Introduction

Neonatal morbidity and mortality remain serious global health concerns. In 2018 alone, approximately 2.5 million children died in their first month of life [1]. The worldwide neonatal mortality rate (NMR) was estimated to be about 18 deaths per 1000 live births [1] with the Sub-Saharan Africa region experiencing the highest burden of these deaths with an estimated NMR of 28 deaths per 1000 live births. In Kenya, the NMR is still high at 22 deaths per 1000 live births with stark disparities across sub-regions and socioeconomic groups [2]. The majority of neonatal deaths in low-income countries occur at home, partly due to traditional health and illness beliefs that influence families’ healthcare-seeking behavior [3].

While major clinical causes of newborn morbidity and deaths are well documented such as neonatal sepsis, pneumonia, tetanus, diarrhea, birth asphyxia and prematurity [4, 5], families’ and communities’ understanding of the etiology of illnesses in newborns and young infants 0–59 days old or sick young infants (SYIs) is not well understood. Studies on this subject have noted that perceptions of causes of illnesses in SYIs may vary across socio-cultural and economic contexts [6–8]. In some communities, newborn and young infant illnesses and deaths are attributed to supernatural forces such as evil spirits or punishment from God [7, 9–11]. Individuals, families, and communities may also attribute illnesses to natural or biomedical causes such as poor environmental and sanitation conditions, personal hygiene, and biological factors [12] as well as social-related reasons such as violation of taboos [13].

Despite the risk of dying being highest in the first month of life, there is limited evidence on communities’ perception of the causes of illnesses in SYIs 0–59 days old in Kenya. There is also limited evidence on whether beliefs about causes of illnesses in SYIs differ across regions. The few existing studies on this subject only focus on a broader age group of children under five years [14] and tend to be limited to specific conditions such as malaria or pneumonia [15–17]. This study explores communities’ understanding of the etiology of illnesses in SYIs in four counties in Kenya. Information on communities’ understanding of the causes of illness in SYIs is key to strengthening interventions and improving newborn health outcomes.

## Data and methods

### Study design

The qualitative data used for this paper were drawn from a formative assessment of a larger study known as the *Ponya Mtoto* project. More details about the project can be found at: https://www.harpnet.org/project/ponya-mtoto/. The *Ponya Mtoto* project aimed at assessing the feasibility, acceptability and sustainability of implementing the World Health Organization (WHO) Guideline for the Management of Possible Serious Bacterial Infection (PSBI) in SYIs 0–59 days old where referral is not feasible in Kenya [18]. The larger, original study used a mixed-method cross-sectional study design involving a health system capacity assessment and health facility assessments in combination with a qualitative exploration based on in-depth interviews (IDIs) and focus group discussions (FGDs). This paper focuses on and reports findings from an aspect of a qualitative study that explored community members’ understanding of the causes of illnesses in SYIs.

### Study setting

The study was conducted in four counties, namely, Turkana, Bungoma, Mombasa, and Kilifi. Table 1 highlights key socio-economic indicators for these counties. The four counties were selected due to their distinct geographical, socio-economic and cultural diversity, and high NMR burden compared to the national average of 22 deaths per 1000 live births. Turkana is an arid and semi-arid region with a predominantly nomadic population dispersed across difficult terrains with limited access to healthcare facilities. Bungoma is largely a rural agrarian economy and predominantly occupied by the Bukusu tribe. Mombasa has a large coastal urban sub-population living in informal settlements faced with persistent health and economic inequalities. Finally, Kilifi represents a coastal region with both rural and urban sub-populations. In each county, the study was conducted in two sub-counties selected in consultation with respective County Health Management Teams (CHMTs). Within each sub-county, 12 facilities stratified by levels (hospitals, health centers and dispensaries) were selected as part of the larger project sites. Participants were drawn from villages surrounding these facilities.

**Table 1:** Key indicators for the study sites.

*Key indicators*	*Bungoma*	*Turkana*	*Kilifi*	*Mombasa*	*National*
Neonatal mortality rate/1000 live births	33/1000	60/1000	26/1000	39/1000	22/1000
Infant mortality rate /1000 live births	97	60	NA	70	39
Under five mortality rate /1000 live births	145	74	141	91	52
Skilled delivery	41.4%	22.8%	52.3%	82.8%	62%
Immunization coverage	70.0%	61.8%	74.1	78.6%	74.6%
Proportion of women 15–49 year with no education	0.9%	64.1%	24.4%	5.8%	7.0%
Population (as at 2019)	1,670,570	926,976	1,453,787	1,208, 333	47,564,296

Source: Kenya Demographic Health Survey 2014/15 [2] and Kenya Population and Housing Census, 2019 [19]

NA = not available

### Study participants

Potential participants were female caregivers, defined here as, a mother, a family member, or a paid helper who regularly looks after a child, and fathers with a SYI (0–59 days) including those who lost a newborn within two months prior to the study date were deemed eligible and recruited to participate. We used purposive sampling and adopted a maximum variation approach based on participant’s age and region. Participants were selected to include 1) a range of caregiver age groups (15–18 years, 19–24 years and 25–45 years) to capture the experiences and views of adolescent, young and older caregivers/mothers regarding newborn and young infant care; and 2) fathers with SYIs aged 18–34 years and 35 or more years to capture the views of young and older men. Respondent selection ensured that each region and age group were represented. Eligible participants were identified in the community with the help of local leaders, mainly community health volunteers (CHVs) and village elders. We preferred to use local leaders as they were knowledgeable about the members of their community and in a good position to locate potential participants.

### Data collection

Data collection was conducted between August and September 2018 and involved IDIs and FGDs (see Table 2). The purpose of the IDIs (total 23 collected) was to understand caregivers’ personal experiences of newborn care, and beliefs as well as care-seeking practices for SYIs 0–59 days old. The interviews explored caregiver’s understanding of causes of illnesses, recognition of danger signs, decision-making and care-seeking practices for SYIs as well as challenges and barriers to accessing SYIs health services including referral. The FGDs (total 32) explored community beliefs on causes of illness and care-seeking practices for SYIs. We had planned to conduct at least two FGDs with fathers (one with a younger group and another with older group) in each site, however, we managed only seven FGDs due to challenges recruiting participants in Bungoma.

**Table 2:** Data collection methods.

Category of interviews	Turkana	Bungoma	Mombasa	Kilifi	Total
**In-depth interviews**					
Very young caregivers 15–18 years	2	3	4	2	**11**
Young caregivers 19–24 years	4	3	2	3	**12**
Total	**6**	**6**	**6**	**5**	**23**
**Focus group discussions**					
Very young caregivers 15–18 years	2	2	1	2	**7**
Young caregivers 19–24 years	4	2	3	3	**12**
Older caregivers 25–45 years	2	2	1	1	**6**
Young fathers 18–34 years	1	0	1	1	**3**
Older fathers 35 or more years	1	1	1	1	**4**
Total	**10**	**7**	**6**	**8**	**32**

Research assistants with a social science background, experienced in qualitative research methods and trained by the study team on protocols and research ethics, conducted the IDIs and FGDs. Data were collected using a guide that was developed in English and translated into Kiswahili (widely spoken in the study areas) and Turkana language (for Turkana sites), and pre-tested in each site in a community outside the study’s sampling frame. The IDIs were conducted in participants’ homes while FGDs comprising of 8–10 participants were held at centralized places convenient for participants away from disturbances (mainly in school and church halls). All FGD participants received reimbursement for transport to and from the discussion venue. Each data collection session lasted 60–90 minutes and was audio-recorded with the consent of the participants.

### Analysis

Audio-recorded interviews were transcribed verbatim, translated to English where necessary. We did not however back-translate transcripts into Kiswahili/Turkana to determine if any meaning was lost in the process. The transcripts were transferred to and analyzed using a qualitative software NVivo version 12 (QSR International Pty Ltd). Data were analyzed using a thematic framework approach—a method for identifying, analysing, and reporting patterns (themes) within data [20]. We specifically used an inductive approach to developing a codebook to guide our thematic analysis [21]. Ten members of the research team, including three of the authors (GO, CN and TA), read at least 2 transcripts from each site to familiarize with the content and obtain a broad overview of the participant’s responses. The transcripts were then annotated by highlighting ideas that were interesting or significant. A thematic framework or codebook of themes and sub-themes was then developed based on these highlights and topics on the guides. All transcripts were reread and coded for themes and sub-themes by four coders trained in qualitative data analysis. Analysis charts were then prepared for each thematic area and category of participants. These charts were used to identify common themes across participants and sites. Analysis for this paper is specifically focused on female caregivers’ and fathers’ understanding of the causes of illnesses in SYIs 0–59 days old. We followed the COREQ criteria in conducting the analysis and interpretation of the results [22].

### Ethical considerations

This study received ethical approval from the African Medical and Research Foundation (AMREF) Ethics and Scientific Review Committee (as ESRC P430/2018) and the Population Council’s Institutional Review Board (as Protocol 838). Written informed consent was obtained from each participant before conducting an interview. Participants aged less than 18 years were considered emancipated minors, defined as individuals who have assumed adult responsibilities, such as household headship, marriage, or procreation [23].

## Results

The results describe mothers’, fathers’, and other caregivers’ perceived causes of illnesses in SYIs which were broadly categorized into two main themes either due to natural (biomedical) or supernatural (traditional) elements and are outlined below.

### Perceived natural causes

Under natural causes, many mothers, fathers, and other caregivers attributed newborn and young infant illnesses to; 1) unfavorable environmental conditions; 2) poor maternal and child nutrition practices; and 3) poor maternal and child healthcare practices. Generally, there were commonalities across sites, age groups and gender regarding perceived natural causes of illnesses in SYIs with no specific variation between the four counties.

#### Unfavorable environmental conditions

Many caregivers across the sites attributed illnesses in SYIs such as respiratory tract infections to exposure to unfavorable environmental conditions. For example, pneumonia was attributed to exposure to cold weather or air.

“*During cold season like now*, *the baby may contract pneumonia which is due to cold; you cannot afford to leave the baby without socks*, *not well covered*…*so*, *you should keep the baby warm to prevent such illnesses…”*[FGD, Caregivers 19–24 years, Bungoma]

*Sometimes the babies are covered too much*. *They sweat then the mothers uncover them and probably forget to cover them again*. *The cold will get to the baby’s chest and then the baby will get pneumonia*. [FGD, Caregivers 15–18 years, Turkana]

Caregivers attributed symptoms such as vomiting, gastrointestinal discomfort and diarrhea to poor hygiene and sanitation practices such as putting on dirty clothes, failure by the mother to keep the breast clean and wash hands before breastfeeding or handling the baby. Most caregivers also noted that a newborn or a young infant may get infections if exposed to a dirty feeding environment, for example, failure to keep the baby’s feeding bottles and other feeding equipment clean or leaving the baby’s food exposed.

*“Babies may get infections from dirt*, *for example*, *if you do not wash the [feeding] bottles or when you do not cover baby’s foods such as milk*, *the child will get sick from [germs]”* [FGD, Caregivers 25–45, Mombasa]

*“If the mother is not taking regular showers or washing her breasts properly*, *the baby may get a stomachache or diarrhea…”* [FGD, Caregivers 15–18 years, Turkana]

*“As a mother*, *you also need to be clean*, *and not coming from wherever with sweat and breastfeeding the baby without cleaning [yourself]*, *it could bring problems to the baby*. *Ensure that the baby’s food is clean”* [IDI, Caregiver 19–24 years, Bungoma]

The perceptions of fathers regarding natural or biomedical causes of illness in newborns and young infants were similar to those of caregivers. As narrated by one participant below, many fathers attributed newborns’ and young infants’ illnesses to poor hygiene and sanitation conditions and exposure to cold environment,

*“Babies get illness due to failure by the mother to observe hygiene when breastfeeding*, *like when the breast is dirty*, *introducing the baby to solid food before six months after delivery*, *failure to ensure the baby is warm*, *failure to ensure the baby is covered well before sleeping at night thus exposing the child to cold weather*.*”* [FGD, Fathers >35 years, Turkana]

#### Poor maternal and child nutrition practices

Poor mother and child feeding practices were considered a major cause of illness in newborns and young infants. Most caregivers noted that feeding a newborn or a young infant food other than breast milk before the recommended age for weaning (six months) could cause illnesses. Most caregivers from across all sites mentioned that failure to breastfeed a baby exclusively during the first six months could cause illnesses.

*“*…*to improve the health of the infant*, *we need to exclusively breastfeed the baby*, *it is our responsibility as mothers*…*Second*, *we need to eat a healthy diet so that the milk can also be healthy for the baby*.*”* [IDI, Caregiver 19-24years, Mombasa]

*“A young baby like mine should not be given solid food because it brings about sicknesses*. *They should be breastfed exclusively for six months before introducing [solid] food*.*”* [IDI, Caregiver 19–24 years, Bungoma]

Participants from Turkana County believed that eating certain wild fruits while breastfeeding, a common occurrence during drought and famine seasons, could harm a newborn or a young infant. Many fathers noted that some wild fruits contain poisonous element which might contaminate breast milk if ingested and indirectly cause illness in a newborn or a young infant. Some wild fruits were also believed to lack essential nutrients for milk production required for the growth and development of a newborn or a young infant.

*“Mothers who feed on wild fruits may cause illness to the baby*. *Some of these fruits do not have any nutritional values for the mother and the baby*. *A mother feeding on wild fruits will lack the required nutrients to produce enough milk for the baby*. *Also*, *the baby will suck milk containing [poisonous] elements of these wild fruits*, *which exposes them to illness*.*”* [FGD, Fathers >35, Turkana]

Some caregivers associated beans with gassiness or fussiness in newborns and young infants.

*“Sometimes the mother eats gassy foods like some types of beans*. *Now when that mother breastfeeds the baby*, *the baby also becomes gassy or gets a stomach-ache*. *The baby will be uncomfortable*, *fidget a lot*, *or even cry*.*”* [FGD, Caregivers, 15–18 years, Turkana]

#### Poor maternal and child healthcare practices

Some newborns’ and young infants’ illnesses were linked to poor maternal and child healthcare practices such as failure to go for antenatal care, giving birth at home, and failure to sleep under insecticide-treated nets. Caregivers noted that giving birth at home might expose newborns to infection, mainly through inappropriate cord care practices such as cutting the cord with an unsterilized razor blade and smearing the cord area with cow dung and handling a newborn without gloves.

*“Normally*, *when you give birth at home*, *the mother-in-law is the one who cuts the placenta using a razor blade and ties with a thread*. *You cannot know the hygiene of the one handling the infant at home*.*”* [IDI, Caregiver 19–24 years, Kilifi]

*“There are things that normally happen when a mother delivers at home with the help of TBA [traditional birth attendant]*. *After cutting the cord*, *they [TBAs] normally smear it with cow dung*, *and you know cow dung is not clean*, *it can cause infection to the baby like tetanus*.*”* [IDI, Caregiver 19–24 years, Bungoma]

Participants also noted that some traditional practices may expose newborns or young infants to unhygienic conditions or inappropriate feeding practices and cause illnesses. For example, some communities in Turkana County do not allow mothers to bathe until after the umbilical cord falls off. Mothers were also not allowed to breastfeed a newborn until after the naming ritual. Instead, newborns are fed on goat milk while they wait to undergo naming rituals.

*“You and your child would not take a bath until that day when the child’s umbilical cord drops*, *it can even take five days without taking a bath*. *In some clans in []*, *they don’t breastfeed the child until the name is given in the morning*. *These [practices] can expose the child to many diseases*.*” [*FGD, Caregivers 19–24 years old, Turkana]

Newborn and young infant illnesses were also linked to overworking during pregnancy. Mothers, fathers and other caregivers noted that doing heavy chores during pregnancy might cause health problems to the mother and her unborn child.

“*The kind of activities and work that they do while pregnant like walking long distances in search of firewood and carrying heavy logs might cause problems to the mother and illnesses for the infants when they are born*.*”* [FGD, Fathers > 35 years, Turkana]

### Perceived supernatural causes

Some of the participants associated newborns’ and young infants’ illnesses to supernatural or traditional practices. While some supernatural or traditional beliefs about causes of illness in SYIs were similar across the study sites, age groups and gender, others were specific to a region, and both are described in the section below.

A common cross-county perception was the belief that symptoms such as gastrointestinal discomfort, inability to breastfeed, rashes, body swellings, fever and persistent crying of newborn or young infants were due to the ‘evil eyes’. The concept of the ‘evil eyes’ was described locally using different terms such as ‘bad eyes’, ‘throwing bones at the baby’ or making some cynical comments about the baby. Many participants believed that conditions caused by the ‘evil eyes’ did not require hospitalization and only treatable by traditional means or through prayers.

*“There are those with evil eyes when they look at your baby it makes them cry throughout due to stomachache…When you take such a baby to the hospital*, *they [providers] will not diagnose any disease*, *but when you take them to pastors or imam they’ll get well*.*”* [IDI, Caregiver 15–18 years, Mombasa]

*“Some people look at the baby and cast bad things on the baby…it is called [Vikumba]*. *They throw bones to the baby and then the body becomes hot and the feet and hands are cold*.*”* [FGD, Caregiver 19–24 years, Bungoma]

*“Some mothers in the village have a habit of bewitching young infants during breastfeeding*. *This makes the child cry and sometimes stops breastfeeding*.*”* [FGD, Fathers <35 years, Turkana]

*“In our community*, *if somebody walks up to you baby and says a statement such as ‘your baby has such a light skin complexion’ or ‘your baby is so fat’*. *Such statements could make the baby sick*.” [IDI, Caregiver 19–25 years, Turkana]

Some traditional beliefs about the causes of newborn and young infant illnesses were context-specific. For example, in some communities in Western Kenya, it is a taboo for a father to talk about a newborn to anyone while traveling.

*“They say that if you go with the baby to a place and the father starts talking about the baby*, *s/he will start to cry and fall sick…[sometimes] they cry and faint…”* [IDI, Caregiver 19–24 years, Bungoma]

In coastal areas such as Mombasa and Kilifi, conditions such as high fever, sunken eyes and convulsions were attributed to a bird, an owl. Some caregivers and fathers believed that the presence of an owl in a compound or on the roof of a house was a sign of a bad omen and sometimes would cause illnesses in young infants.

*“We believe that there is a bird that sometimes makes the baby fall sick*. *When the baby has a high fever and shows certain looks such that the baby just stares up*, *it’s because of this bird*, *called the owl*.*”* [FGD, Young caregivers 19–24, Kilifi]

*“Most of the time we have observed that when a child has been born*, *the owl comes either on top of the roof or at the corner of the house*. *The child’s black part of the eyes [pupil] gets lost*, *it becomes white*.” [FGD, Fathers <35 years, Mombasa]

Some illnesses in SYIs were associated with the naming of the baby. For instance, persistent crying of a young infant was associated with the perception that the baby did not like the name given or the ancestral spirits were unhappy with the baby’s given name.

*“There are some illnesses that come from the family name*. *For example*, *if I give a child a name from my family side and the father wants the baby to be named after his family*, *that can cause the child to be sick*…*the baby will cry a lot*.*”* [FGD, Caregivers 25–45 years, Bungoma]

Other newborn and young infant illnesses were linked to extramarital affairs.

*“… when the father of the baby comes back after having extramarital sex and carries the baby*, *the baby will get weak and become sick most of the time*.*”* [FGD, Caregivers 19–24, Mombasa]

*“When a woman gets another man and sleeps with him*, *that person's sperms are different from the baby’s father sperms*, *therefore may cause diseases to the child*.*”* [FGD, Caregivers 19–24, Turkana]

Some fathers, especially older men from western Kenya, believed that using contraceptives was harmful to the mother’s or newborn’s health.

*“I have been comparing my first child*, *and the second born*. *For my first child*, *the mother had never used family planning*, *and this child never showed any signs of sickness*. *However*, *this second one has been sickling*. *If you listen to what my colleagues are saying*, *there is only one problem*. *So according to me [contraceptives] is the problem affecting our children’s health*.*”* [FGD, Fathers > 35 years, Bungoma]

## Discussion

We explored communities’ understanding of the etiology of illnesses in SYIs in 4 counties in Kenya. The findings showed a wide range of communities’ beliefs about the causes of illnesses in SYIs that were similar across the counties and others that were unique. Generally, the perceived causes of diseases and illnesses in SYIs were dichotomized as either due to natural (biomedical) or supernatural (traditional) reasons. The findings are consistent with Murdock’s ill-health causation framework which distinguishes between beliefs about the medical and non-medical causes of illness and disease [24].

There were commonalities across counties and age groups in the perceived natural causes of illnesses in SYIs. These beliefs were summarized into three: 1) unfavorable environmental conditions; 2) poor maternal and child nutrition practices; and 3) poor maternal and child healthcare practices. Symptoms such as diarrhea and vomiting were attributed to poor sanitation and hygiene conditions. The results are similar to the findings of a study conducted in Amhara region in Ethiopia where caregivers attributed newborn illnesses to gaps in hygiene practices or unmet maternal nutritional needs [12]. Some mothers, fathers and other caregivers associated pneumonia with exposure to cold environment/air. A similar finding have been noted in qualitative studies conducted in Ghana and Nigeria [25, 26]. While illness conceptualization play an important role in the decision regarding treatment options [27, 28], knowledge of biomedical causes alone may not lead to healthcare-seeking from facilities in settings where traditional beliefs are pervasive [29, 30]. There is need for interventions to reinforce correct beliefs about causation and treatment of illnesses in SYIs to promote care-seeking from health facilities.

Perceived supernatural causes of illnesses in SYIs were pervasive across the study sites and mainly revolved around traditional beliefs and childcare practices. Some of these beliefs were common across sites, age groups and gender. For example, many mothers, fathers and other caregivers across the sites attributed symptoms such as fever, gastrointestinal discomfort, changes in skin color, inability to breastfeed, and excessive crying to the ‘evil eye’, a concept described using different terminologies in the local settings. The findings also show that some of the perceived supernatural causes of illnesses in SYIs were geographically unique. For instance, the belief that some illnesses in SYIs were caused by a bird (owl) or someone talking about an infant while traveling was noted among coastal and western communities, respectively. Erroneous beliefs about causes of illnesses in SYIs such as linking illnesses to a child’s given name were also common. Our findings are similar to studies conducted elsewhere in low- and middle-income countries where illnesses or death to children have been attributed to the ‘evil eye’ or malevolent spirits [7, 10, 29–31]. A study in the Ashanti Region of Ghana revealed how caregivers attributed pregnancy loss and newborn mortality to a local illness called *asram*–believed to be caused by evil spirits [11].

Beliefs about causes of illness in SYIs, whether biomedical or supernatural, were similar along gender lines. However, some erroneous beliefs were more pronounced among fathers than female caregivers. For example, some fathers attributed illnesses in SYIs to contraceptive use. The variation in knowledge about causes of illnesses in SYIs between men and women have been explained in the literature. Studies suggest that female caregivers are more likely to be aware of illnesses in SYIs than men due to their greater interaction with the healthcare system during antenatal care, postnatal care, immunizations, and family planning clinics among other activities [29, 32].

An important finding from our study is that, sometimes, the beliefs about biomedical causes co-exist with supernatural beliefs. Although we did not explore the category of beliefs about causes of illness in SYIs that were given more weight, most caregivers and fathers believed in both biomedical and supernatural causes. The plurality in the attribution of causes of illness in SYIs has been reported in other studies in sub-Saharan Africa. For example, in a study in Uganda, caregivers attributed symptoms of severe malaria or other biomedical illnesses to traditional and biomedical causes [31]. The findings also compare with results from other studies on ethnoetiology of illness in older children, including evidence from Kenya [14, 15, 33]. A study by Abubakar and others in rural coastal Kenya noted that caregivers expressed multiple parallel beliefs on causes of illness in children under-five years [14]. The findings highlight the complexity of how to reinforce communities’ understanding of biomedical causes of illnesses and improve care-seeking behaviour. There is need for healthcare providers to be cognizant of medical and non-medical beliefs in the communities they serve as some caregivers may prefer to use alternative or traditional remedies, and only turn to biomedical treatment if symptoms persist or the condition becomes severe.

The findings have important program implications. Evidence shows that beliefs about disease etiology influence care-seeking behavior [34, 35]. In our study, participants preferred traditional remedies to biomedical care for illnesses that were attributed to supernatural causes. Many participants believed that illnesses in SYIs caused by the ‘evil eye’ did not require hospitalization and can only be managed through traditional treatment means such as traditional healers, herbal medicines, or prayers. Such beliefs may hinder or cause delays in seeking healthcare for SYIs from a health facility, thus leading to worse newborn health outcomes including death. The findings underscore the need for interventions to address the gap between local beliefs about causes of illnesses in SYIs and care-seeking from health facilities to improve newborn health outcomes.

The findings have also implications on the adoption and implementation of newborn and young infant focused policies and guidelines such as the new WHO’s PSBI guidelines [18]. Efforts to implement WHO’s PSBI guidelines and other newborn-focused interventions in low-resource settings should integrate local understanding of disease causations to increase demand for SYIs services from health facilities. Recent evidence on the feasibility of implementing PSBI guidelines in low-income countries have demonstrated the need for social and behavior change interventions targeting families and communities to improve care-seeking from health facilities [36, 37]. Strengthening community health structures by involving community health volunteers in creating awareness on the causes of illnesses and early identification in the community and referral of SYIs cases can be effective in improving community knowledge on the causes of illness in SYIs and improving care-seeking from health facilities.

## Study strengths and limitations

Our study participants were drawn from a wide geographical area representing diverse socio-economic and cultural settings and include caregivers and fathers with SYIs from a range of age groups. These elements create the diversity of the sample and enable identification of similarities and differences in beliefs. However, the study is not without limitations. First, as in any other qualitative studies, the study findings cannot be generalized to the general population. Second, we were only able to interview female caregivers and fathers with SYIs. Third, the analysis does not take into consideration participant’s education level which could influence their knowledge of causes of illness in newborns and young infants. Fourth, participants were purposively identified with the help of community leaders who are known to them, which could lead to selection bias. There is also a potential of social desirability bias that might have influenced responses. Despite these limitations, the study provides useful information on communities’ perception of causes of illnesses in SYIs 0–59 days old which could inform newborn and young infant health interventions in Kenya and other similar settings.

## Conclusion

There were commonalities in perceived biomedical causes of illness in SYIs across sites, age groups and gender. Some supernatural beliefs about causes of illness in SYIs such as malevolent spirits were also common across counties while others were unique such as the belief that owls cause illnesses in SYIs among coastal communities. Our findings underscore the need for child health programmes to take into consideration communities’ beliefs and practices regarding disease and health to improve newborn health outcomes.

## List of abbreviations and acronyms

CHVs: Community Health Volunteers
FGDs: Focus Group Discussions
IDIs: In-depth Interviews
NMR: Neonatal Mortality Rate
PSBI: Possible Serious Bacterial Infection
SYIs: Sick Young Infants
WHO: World Health Organization
